# Combined ultrasound and low temperature pretreatment improve the content of anthocyanins, phenols and volatile substance of Merlot red wine

**DOI:** 10.1016/j.ultsonch.2023.106636

**Published:** 2023-10-07

**Authors:** Qi Xie, Yurou Tang, Xueyan Wu, Qingyan Luo, Wentong Zhang, Hanyang Liu, Yulin Fang, Xiaofeng Yue, Yanlun Ju

**Affiliations:** a College of Enology, Northwest A & F University, Yangling 712100, Shaanxi, China; b College of Food Science and Engineering, Northwest A & F University, Yangling 712100, Shaanxi, China; c Heyang Viti-viniculture Station, Northwest A & F University, Yangling 712100, Shaanxi, China

**Keywords:** Ultrasound, Low temperature, Anthocyanins, Phenols, Volatile, Fermentation

## Abstract

Ultrasound combined with low temperature treatment is a new food processing technology. In this study, low temperature, three ultrasound power levels, and their combinations were adopted in the must before fermentation to study their effects on Merlot red wine. The results showed that ultrasound combined with low temperature pretreatment increased the total and monomer contents of anthocyanins and phenols, affected the color of the wine, and significantly increased its antioxidant capacity. In particular, 240 W of ultrasound combined with low temperature pretreatment reduced the bad odors (caprylic acid, benzaldehyde, and 1-ethanol content) and improved the flower and fruit aroma (1-octanol and phenethyl acetate), as well as the aftertaste, thus improving the quality of the wine. Ultrasound combined with low temperature pretreatment positively affected the quality of Merlot red wine.

## Highlights

• Evaluate the effect of ultrasound combined with low temperature pretreatment on wine.
• Pretreatment could increase the contents of anthocyanins and phenols.
• Pretreatment affected the color of wine.
• Pretreatment significantly improve its antioxidant capacity.
• Pretreatment could affect the volatile components and improve the aftertaste.

## Graphical abstract

**Figure:**
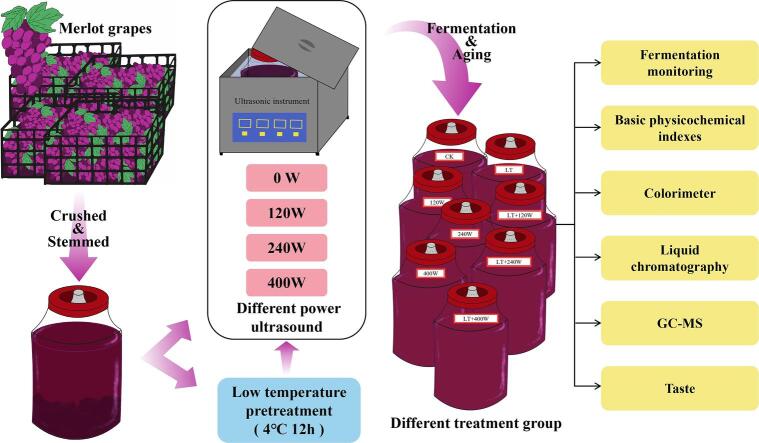

## 1 Introduction

Ultrasound is an advanced non-thermal food-processing technology as an alternative to, or an adjuvant method for, conventional processing techniques [1]. The mechanism of ultrasound extraction of bioactive compounds is the union of multiple physical mechanisms (fragmentation, erosion, sonocapillary effect, sonoporation, local shear stress, and detexturation) [2]. Ultrasound affects food processing by provideing promoting or damage effects on enzyme activities at the molecular level [3]. Ultrasound processing, whether used alone or in combination with other methods, improves food quality significantly, and thus is considered beneficial [4]. In recent years, ultrasound technology has been applied to the processing of food by-products, including fruits [5], vegetables [6], and juices [7]. Gambacorta et al., [8] demonstrated that ultrasound in winemaking increased the extraction of polyphenols from grapes and improved the sensory characteristics and health benefits of wine due to a higher content of nutraceuticals. Natrella et al., [9] applied an ultrasound treatment during the vinification of three different red grape cultivars with the aim of assessing the impact on the volatile profile of the wines. A clear separation was observed between the control and ultrasound-treated wines for all three cultivars, with ethyl decanoate, ethyl isopentyl succinate, and butyric acid having the highest discriminant coefficients. Lukić et al., [10] found that high power ultrasound modified phenolic and volatile composition, with greater effect in white wines with lower concentration of antioxidants. Similarly, Aragón-García et al., [11] proposed that application of ultrasound during the pre-fermentative maceration process favors the release and extraction of volatile compounds in greater amounts.

Low temperature pretreatment is now used in various fields of food processing. Low temperature pretreatment is defined as lowering the temperature of foodstuffs reduces microbiological and biochemical spoilage by decreasing microbial growth rates and enzymatic activity [12]. Fruits, vegetables and other foods are processed at temperatures above freezing but below room temperature, around 0 − 5℃. The application of pretreatment at low temperature before fermentation is one of the most applied and investigated practices [13]. The vinification technique with low temperature pretreatment—referred to as pre-fermentative cold maceration—is used to enhance anthocyanin diffusion from the skins to the must, increasing pigment extraction. Casassa et al., [14] found that a short period of low temperature pretreatment before fermentation did not affect the basic physicochemical indexes, volatile compounds, or improve the aroma of Merlot wine, but effectively reduced the astringency and pain perception in the generated wine. Studies have shown that, during pre-fermentative cold maceration of Merlot red wine, an increase in total polyphenol index, anthocyanins, and tannins of around 10 % takes place, and this increment is maintained until the end of the alcoholic fermentation [15]. Yan et al., (2018) used a low temperature technique combined with ultrasound to treat raw sweet white wine and obtained a more microbially stable and higher quality sweet white wine. There are currently no reports on the combined use of ultrasound and low temperature pretreatment in dry wine.

Phenolic compounds are the most abundant fraction of the nonvolatile compounds of red wine and have a large contribution to color, astringency, and bitterness [16]. Moderate amounts of polyphenols in wine are associated with anti-inflammatory activity, endothelial function, and flow-mediated dilation, and thus are beneficial to human health [17]. Anthocyanins are a type of polyphenol derivative, and the copigmentation of anthocyanins is beneficial to the stability of wine color [18]. Wine aroma is a complex mixture of volatile substances that are a crucial determinant of wine quality [19]. The composition and content of volatile compounds lead to a variety of aromas and create differences between wines.

Therefore, a combination of ultrasound and low temperature pretreatment might be a promising method for improving the winemaking process and the quality of wine. With the development of modern food technology, consumer demands for food safety and green approaches have greatly increased. Compared with chemical methods, a physical method is more in line with the requirements of green food. This study aims to fill a knowledge gap in the combined effects of ultrasound and low temperature pretreatment on a red dry wine, which can provide a new direction for the development of wine production technology.

## 2 Materials and methods

### 2.1 Wine samples and vinification process

Grapes (*Vitis vinifera* L*.* cultivars Merlot) were collected from a commercial vineyard located at Yangling, Shaanxi, China (39°54′ N, 116°23′ E) and the sugar content was 191.84 g∙L^-1^. A small container brewing method was used, referring to Jiang et al. [20]. The grapes were sorted by hand, then crushed and destemmed. Pectinase (Lallzyme EX, Lallemand, France) was added at 30 mg∙L^-1^ and SO_2_ was sulfited at 50 mg∙L^-1^. About 4 L (80 % of fermenter) musts were homogeneously distributed in eight 5-L glass fermenters that were cleaned and dried in advance, and were pretreated using one of the following methods: 1) no ultrasound treatment and low temperature pretreatment (CK); 2) 120 W ultrasound treatment (120U); 3) 240 W ultrasound treatment (240U); 4) 400 W ultrasound treatment (400U); 5) no ultrasound treatment but low temperature pretreatment (LT); 6) 120 W ultrasound treatment and low temperature pretreatment (LT + 120U); 7) 240 W ultrasound treatment and low temperature pretreatment (LT + 240U); or 8) 400 W ultrasound treatment and low temperature pretreatment (LT + 400U). The ultrasonic device is ultrasonic bath (KQ-400DE, Kun Shan Ultrasonic Instruments Co., Ltd, China). The internal temperature of the ultrasonic device was controlled to be constant at room temperature (25℃ ± 1℃) by continuous injection of cold water and discharge of hot water. The ultrasound treatment time was 30 min and the low temperature pretreatment was at 4 °C for 12 h.

The pretreated musts were inoculated with yeast (*Saccharomyces cerevisiae* RC212, Lallenmand, Denmark) that had been activated by grape juice at 0.5 g∙L^-1^ in terms of dry weight. The fermentation temperature was 21 °C ± 2 °C. During alcoholic fermentation, we monitored the temperature, specific gravity, and sugar degree each day. The wine was racked and decanted after 2 d of fermentation. When the specific gravity became constant, the wines were racked and 60 mg∙L^-1^ of SO_2_ was added to complete the fermentation. All analyses were performed after cold-stabilization for 60 d [21] at 0 °C ± 2 °C and maturation for 60 d in bottles at 20 °C.

### 2.2 Chemicals

Methanol, ethyl acetate, acetic acid, and acetonitrile (HPLC grade) were obtained from Sigma (Shanghai, China). Folin–Ciocalteu, 6-hydroxy-2,5,7,8-tetramethylchroman-2-carboxylic acid, and 2,2-diphenyl-1-picrylhydrazyl (analytical grade) were obtained from Fisher (Suwanee, GA, USA). The standards (citric acid, tartric acid, malic acid, amber acid, lactic acid, acetic acid, delphinidin 3-O-glucoside, cyanidin 3-O-glucoside, petunidin 3-O-glucoside, peonidin 3-O-glucoside, malavidin 3-O-glucoside, peonidin 3-O-(6-O-acetyl)-glucoside, malavidin 3-O-(6-O-acetyl)-glucoside, *trans*-peonidin 3-O-(6-O-p-coumaryl)-glucoside, *trans*-malvidin 3-O-(6-O-p-coumaryl)-glucoside, gallic acid, protocatechuic acid, catechin, gentianic acid, caffeic acid, epicatechin, p-coumaric acid, rutin, *trans*-ferulic acid, rhizoside, resveratrol, quercetin, and kaempferol) used in this study were of HPLC grade and obtained from Sigma (Shanghai, China).

### 2.3 Detection of basic physicochemical indices of wine samples

The detection of the ethanol, pH, volatile acid, and titratable acid of the wine samples was conducted according to International Vine and Wine Organization [22]. The total phenolic content (TPC) was determined using the Folin-Ciocalteu assay reference to Jara-Palacios et al., [23]. The total anthocyanin content (TAC) was determined using pH-differential spectrophotometry based on the method of Cliff, King, and Schlosser [24] with some modifications. The total flavonoid content (TFC) was determined using aluminum chloride colorimetry [25]. Total flavan-3-ol (TFOC) was determined using spectrophotometric determination [26].

### 2.4 Color intensity of the wine samples

The a* (green/red), b* (blue/yellow), and c* (saturation) values and h° (hue angle) were obtained directly by analyzing the color of wine samples with a wine color analyzer (W100, Hanon Advanced Technology Group Co., Ltd, China). An equal amount of liquor was added to a standard wine glass and photographed in the same small studio to obtain a true image of the color of the wine sample.

### 2.5 HPLC analyses of the wine samples

#### 2.5.1 Sugar

The sugar analysis was based on published reports (Ju et al., 2018). The wine samples were filtered with a 0.45-μm filter membrane and then injected into a 1.5-mL bottle with a sterile syringe. A high performance liquid chromatography (HPLC) system (Agilent 1100, Agilent Technologies Co. Ltd., USA) was used for the determination of sugar. Chromatographic conditions were as follows: a ZORBAXSB-C18 (4.6 mm × 250 mm, 5 μm) was used as the column; the mobile phase was acetonitrile–water (7525, V/V); the flow rate was 1.0 mL/min; the column temperature was 40 °C; difference detection was used; the sample size was 10 μL; and the analysis time was 20 min. The curve was drawn with standards and compared with the standard curve to obtain the results.

#### 2.5.2 Organic acid

The organic acid analysis was based on that of a previously published report [27]. The wine samples were filtered with 0.45-μm filter membranes and then injected into 1.5-mL bottles with sterile syringes. An HPLC system (Agilent 1100, Agilent Technologies Co. Ltd., USA) was used for the determination of organic acid. The chromatographic conditions were as follows: a Hypersil GOLDa Q (250 mm × 4.6 mm, 5 μm) was used as the column; the mobile phase was 0.01 mol/L dipotassium phosphate solution (pH 2.2); the flow rate was 0.5 mL/min; the column temperature was 25 °C; the sample size was 10 μL; the wavelength was 210 nm; and the analysis time was 30 min. The curve was drawn with standards, and the quantitative calculations were carried out according to the curve of standard samples.

#### 2.5.3 Anthocyanins

Anthocyanin identification was performed following a modification of the method described by Yue et al., [28]. After filtering through a 0.22-μm filter, the anthocyanin content was analyzed by an HPLC system (LC-20AT, Shimadzu Co., Ltd., Japan) fitted with a C18 column (4.6 × 250 mm, 4 μm, SynergiTM 4-µm Hydro-RP 80A, Phenomenex, USA). Formic acid–water-acetonitrile (1:32:4, v/v/v) was used as solvent A, and formic acid–water-acetonitrile (1:16:20, v/v/v) was used as solvent B. The solvent gradient elution was as follows: 0.00–15.00 min, 0–10 % B; 15.00–30.00 min, 10–20 % B; 30.00–45.00 min, 20–35 % B; 45.00–46.00 min, 35–100 % B; and 50.00–51.00 min, 100–0 % B. The standard product was dimethoxylin-3-O-glucoside. The standard was used to establish a calibration curve, and the relative concentration of each monomeric anthocyanin was expressed as the equivalent of dimethoxylin-3-O-glucoside.

#### 2.5.4 Phenolic substances

The extraction of phenolic substances in wine samples was according to reports [29] with some modifications. HPLC was used for the phenolic substance content determination. Samples were filtered through a 0.22-μm filter, in an LC-20AT (Shimadzu Co., Ltd., Japan). Formic acid–water (1:99, v/v) was used as solvent A, and acetonitrile (HPLC grade) was used as solvent B. The solvent gradient elution was as follows: 0.00–5.00 min, 3–10 % B; 5.00–15.00 min, 10–15 % B; 15.00–33.00 min, 15–26 % B; 33.00–40.00 min, 26–45 % B; and 40.00–50.00 min, 45–3 % B. Mixed standards of five concentration levels were prepared and standard curves were drawn. The identification and quantification of phenolic substances were undertaken according to the calibration curve of the respective standard.

### 2.6 Analyses of the volatile compounds of wine samples

Volatile compounds were analyzed by a gas chromatography-mass spectrometry system (GC–MS) (ThermoFinnigan, USA) using a published methodology [30] with some modifications. The GC conditions were: a DB-WAX column (30 m × 0.25 mm, 0.25 µm) was used; helium (He) was the carrier gas; the flow rate was 1 mL/min; the column temperature was maintained at 40 °C for 3 min, then increased to 160 °C at 4 °C/min, and then to 230 °C at 7 °C/min for 8 min; and the temperature of the connecting rod was set to 230 °C. The MS conditions were: full scan, ranging from 33 to 450 amu, once per second; electron bombardment was the ion source; the ion source temperature was 230 °C; the electron energy was 70 eV; the filament current was 0.2 mA; and the detector voltage was 350 V. Volatile compound identification was conducted according to the NIST 14 mass spectrum library and retention indices of the authentic standards. The internal standard was 4-methyl-2-amyl alcohol (1.0 g/L). The calibration curve of volatile compounds was established by using the area ratio and concentration ratio of the target compound and internal standard. The volatile compounds were quantitatively analyzed using calibration curves.

### 2.7 Analysis of the antioxidant capacity of wine samples

The antioxidant activity of wine samples was determined using the methods of 1,1′-diphenyl-2-picryl-hydrazyl radical (DPPH) and ferric-reducing antioxidant power (FRAP). The DPPH method referred to previous methods [31]. Three replicates of each sample were prepared. Briefly, 0.1 mL of a wine sample was added to 3.9 mL of the DPPH mother liquor (25 mg∙L^-1^ of a DPPH formic acid solution) and mixed evenly, and the light absorption value was determined at 517 nm after 20 min of a light-shielded reaction. The same volume of a 15 % ethanol solution was used to replace the wine samples as the control. The DPPH value was expressed as DPPH (%) = [(A_blank_ – A_sample_)/A_blank_] × 100 % [32].

The FRAP method referred to previous methods [33] with some modifications. Briefly, 1 mL of a wine sample was reacted with 5 mL of tripyridyltriazine (TPTZ) in a bath of 37 °C for 10 min, and then the absorbance was measured at 593 nm. The results were expressed as FeSO_4_ equivalence (μmol∙L^-1^).

### 2.8 Sensory evaluation of the wine samples

The American Wine Society (AWS) wine tasting list was used with slight adjustments to organize a sensory tasting panel of eighteen people (eight men and eight women). Prior to the tests, the panelists were informed about the procedures, samples, and treatments, and written consent was obtained. In particular, the tasting group was intensively trained for taste and smell using the standard taste and aroma substances of wine. The eight wine samples were blind tasted and scored for degree of clarification, color, aroma balance, aroma intensity, taste balance, flavor intensity, and aftertaste persistence, and the full score of each parameter was 5 points. The parameter ratings were then averaged.

### 2.9 Statistical analysis

The experimental data were analyzed by Microsoft Office 2016 software and SPSS Statistics 22.0 software (Duncan test). The significance level of all test results was α = 0.05. GraphPad Prism 9 software was used to draw line charts and bar charts. After the calculation of the volatile substance normalization, Origin software was used to draw the heat map and perform principal component analysis (PCA).

## 3 Results and discussion

### 3.1 Fermentation process

**Fig. 1:**
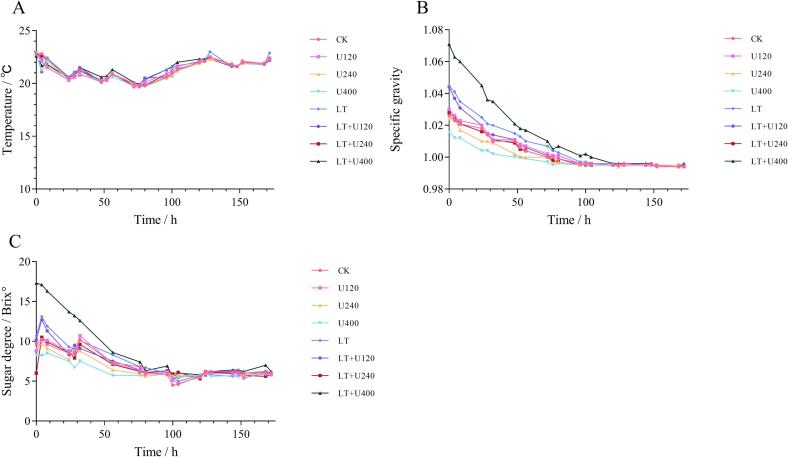
Effects of ultrasonic combined with low temperature pretreatment on fermentation process of Merlot wine. (A): Temperature, (B): Specific gravity, (C): Sugar degree, CK: No-low temperature pretreatment, U120: 120 W ultrasound treatment, U240: 240 W ultrasound treatment, U400: 400 W ultrasonic treatment, LT: low temperature pretreatment, LT + U120: low temperature pretreatment and 120 W ultrasound treatment, LT + U240: low temperature pretreatment and 240 W ultrasound treatment, LT + U400; low temperature pretreatment and 400 W ultrasound treatment.

The changes in temperature, specific gravity, and sugar degree of the wine samples were monitored during fermentation, and the results are shown in Fig. 1. The overall temperature fluctuation trend was consistent, which impacted fermentation kinetics [34], and was primarily dependent on the fermentation process rather than the pretreatment. Due to the different pretreatments, the initial proportion of each group was different. Specific gravity is mainly related to the concentration of the extract in the liquid. It was clearly observed that after the highest power ultrasound treatment and low temperature pretreatment, the extract in grape juice had the highest specific gravity. When no ultrasound treatment was performed, the difference in specific gravity between samples was 1.85 % whether or not low temperature pretreatment was performed. When low temperature pretreatment was not performed, the maximum power ultrasound treatment decreased the specific gravity. With the highest power ultrasound pretreatment, the specific gravity of the low temperature pretreatment samples was 5.41 % higher than that without low temperature pretreatment. In conclusion, the combined ultrasound and low temperature pretreatment increased the concentration of extracts in the pre-fermentation liquid. After fermentation, the proportion in the LT + U400 group decreased the fastest but reached an equilibrium value 24 h later than the other groups. The law of sugar degree value is similar to that of specific gravity, and the same LT + U400 group had the fastest decrease in the sugar degree. It should be noted that small fluctuations in the specific gravity and sugar degree curves may be due to sampling errors caused by an uneven distribution of liquor during fermentation.

### 3.2 Physicochemical characteristics

The physicochemical characteristics of wine, including pH, ethanol, volatile acid, titratable acid, citric acid, tartaric acid, malic acid, amber acid, lactic acid, TPC, TAC, TFC, and TFOC were measured in each sample, and the results are shown in Table 1. There was almost no difference in pH or the content of various acids among the groups, and the values were all within the range prescribed by OIV [22], thus meeting the standard. The ethanol contents of the non-ultrasound treatment groups were higher than that of the ultrasound treatment groups, regardless of whether low temperature pretreatment was performed, which was consistent with previously reported experimental results [35]. The 400 W ultrasound and low temperature pretreatment significantly reduced the alcohol content. The pre-fermentation sugar concentration in the LT + 300 W group was higher than that in the other groups, but the post-fermentation ethanol was significantly lower than that in the control group, possibly because more sugars were converted to other byproducts, such as glycerol [36] and higher alcohols [37]. The TPC of LT + U400 was significantly higher than that of the other groups, and the TPC of the group treated with the highest ultrasound power was higher than that of the corresponding control group with or without low temperature pretreatment. The TAC of the LT + U400 and LT + U400 groups was significantly higher than that of the other groups. Under the same temperature pretreatment conditions, TAC regularly decreased and then increased with an increase in ultrasound frequency. TFC and TFOC showed a similar pattern, with group U400 having the highest levels, and were significantly higher than the control group. Because the TFC of the LT + U400 group was lower than that of the LT group and the flavanols were thought to be chiefly responsible for astringency in wines [38], high power ultrasound may counteract the astringent feeling caused by low temperature pretreatment. A significant interaction was observed between pretreatment and TPC, TAC, TFC, and TFOC. Tang et al., (2016) conducted ultrasound treatment on wine during fermentation, and the results of the physicochemical characteristics obtained were similar to those in this experiment.

**Table 1:** Effects of ultrasound combined with low temperature pretreatment on basic physicochemical indexes of Merlot wine.

	Ultrasonic power								
	No-low temperature pretreatment					Low temperature pretreatment			
	0 W	120 W	240 W	400 W		0 W	120 W	240 W	400 W
pH	3.55± 0.02 cd	3.53± 0.00de	3.58± 0.01ab	3.57± 0.01bc		3.52± 0.00e	3.59± 0.00a	3.59± 0.01ab	3.56± 0.01c
Ethanol / %vol	10.67± 0.06a	10.45± 0.04ab	10.44± 0.01ab	10.46± 0.01ab		10.73± 0.04a	10.48± 0.23ab	10.56± 0.42a	10.06± 0.01b
Volatile acid / g·L^-1^	0.34± 0.02bc	0.34± 0.01bc	0.42± 0.00a	0.31± 0.01c		0.34± 0.01bc	0.33± 0.00bc	0.33± 0.02bc	0.34± 0.01b
Titrable acid / g·L^-1^	5.08± 0.05c	5.49± 0.01a	5.30± 0.04b	5.40± 0.04ab		5.39± 0.03ab	5.11± 0.05c	5.12± 0.07c	5.39± 0.01b
Citric acid / g·L^-1^	0.78± 0.02a	0.77± 0.03a	0.77± 0.04a	0.75± 0.01a		0.80± 0.00a	0.80± 0.00a	0.80± 0.00a	0.79± 0.01a
Tartric acid / g·L^-1^	1.12± 0.03a	1.11± 0.01a	1.12± 0.03a	1.10± 0.00a		1.13± 0.02a	1.12± 0.00a	1.11± 0.02a	1.11± 0.02a
Malic acid / g·L^-1^	2.27± 0.11a	2.20± 0.00a	2.27± 0.11a	2.20± 0.00a		2.27± 0.11a	2.20± 0.00a	2.20± 0.00a	2.20± 0.00a
Amber acid / g·L^-1^	3.32± 0.01a	3.28± 0.18a	3.35± 0.08a	3.41± 0.00a		3.29± 0.01a	3.22± 0.10a	3.22± 0.12a	3.27± 0.02a
Lactic acid / g·L^-1^	3.03± 0.00a	3.00± 0.25a	3.10± 0.10a	3.17± 0.00a		3.03± 0.00a	2.90± 0.09a	2.91± 0.11a	2.99± 0.06a
Total phenol content / mg·L^-1^	98.41± 3.47d	90.71± 3.95e	128.69± 1.18b	119.19± 2.35c		100.46± 0.77d	82.76± 1.33f	86.09± 1.94ef	152.55± 1.18a
Total anthocyanins content / mg·L^-1^	56.11± 1.16b	48.91± 1.57d	50.67± 1.30 cd	55.37± 1.62b		57.72± 3.14b	54.34± 1.45bc	65.21± 3.60a	68.30± 1.25a
Total flavonoids content / mg·L^-1^	607.85± 12.30b	493.62± 5.77 cd	437.46± 1.92d	793.23± 40.77a		785.92± 29.62a	751.69± 37.69a	746.69± 10.38a	554.77± 44.62bc
Total flavan-3-ol content / mg·L^-1^	15.94± 0.21 cd	13.46± 0.26e	17.42± 0.02ab	18.31± 1.47a		9.40± 0.29f	15.16± 1.45d	13.07± 0.17e	16.62± 1.28bc

Note: Different letters in the row indicate significant differences (Duncan test, P ≤ 0.05) among treatments.

### 3.3 Anthocyanin profile

**Fig. 2:**
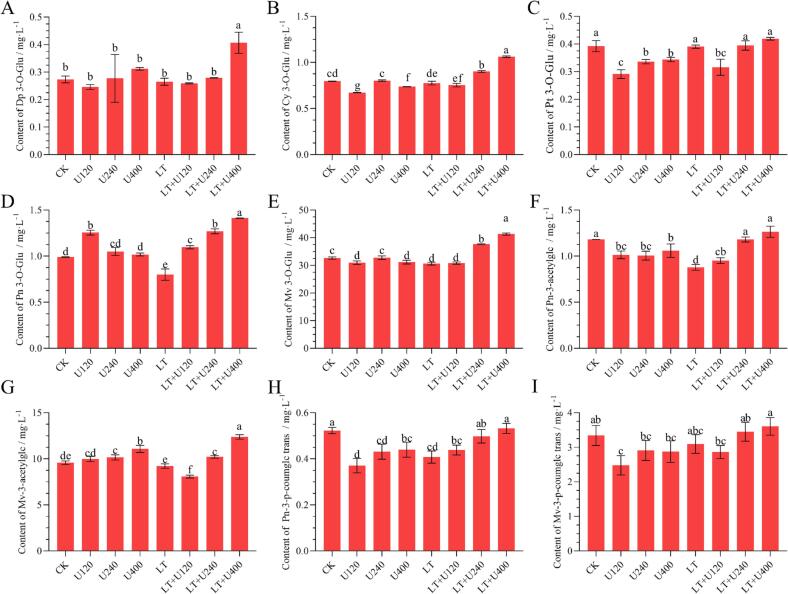
Effects of ultrasonic combined with low temperature pretreatment on anthocyanin of Merlot wine. (A): Delphinidin 3-O-glucoside, (B): Cyanidin 3-O-glucoside, (C): Petunidin 3-O-glucoside, (D): Peonidin 3-O-glucoside, (E): Malavidin 3-O-glucoside, (F): Malavidin 3-O-(6-O-acetyl)-glucoside, (G): Peonidin 3-O-(6-O-acetyl)-glucoside, (H): Trans-Peonidin 3-O-(6-O-p-coumaryl)-glucoside and (I): Trans-Malvidin 3-O-(6-O-p-coumaryl)-glucoside. Values presented are means ± SD (n = 3). Different letters indicate significant differences among treatments using Duncan test (p ≤ 0.05). CK: No-low temperature pretreatment, U120: 120 W ultrasound treatment, U240: 240 W ultrasound treatment, U400: 400 W ultrasonic treatment, LT: low temperature pretreatment, LT + U120: low temperature pretreatment and 120 W ultrasound treatment, LT + U240: low temperature pretreatment and 240 W ultrasound treatment, LT + U400; low temperature pretreatment and 400 W ultrasound treatment.

**Fig. 3:**
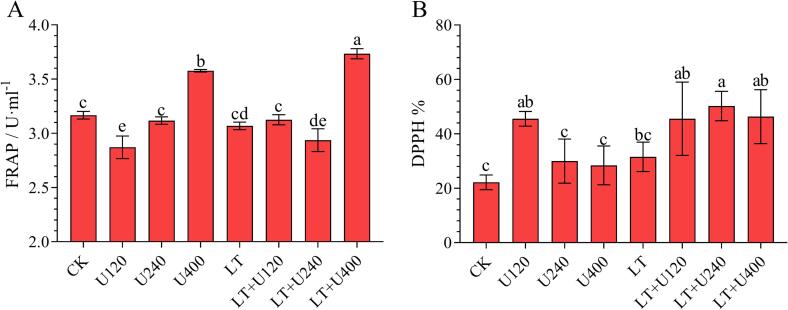
Effects of ultrasonic combined with low temperature pretreatment on antioxidant capacity of Merlot wine. (A): ferric reducing antioxidant power (FRAP), (B): 1,1-diphenyl-2-picrylhydrazyl (DPPH). CK: No-low temperature pretreatment, U120: 120 W ultrasound treatment, U240: 240 W ultrasound treatment, U400: 400 W ultrasonic treatment, LT: low temperature pretreatment, LT + U120: low temperature pretreatment and 120 W ultrasound treatment, LT + U240: low temperature pretreatment and 240 W ultrasound treatment, LT + U400; low temperature pretreatment and 400 W ultrasound treatment.

Anthocyanins are the main pigments of red grapes primarily responsible for the color of red wines [39], and therefore play a major role in consumer preference and appreciation [40]. Fig. 2 shows the effect of ultrasound combined with low temperature pretreatment on the anthocyanin content of Merlot wines, expressed as the means (mg·L^-1^) of the three analytical replicates. Nine anthocyanin compounds were identified and quantified: Dp-3-O-Glu, Cy-3-O-Glu, Pt-3-O-Glu, Pn-3-O-Glu, Mv-3-O-Glu, Pn-3-acetylglc, Mv-3-acetylglc, Pn-3-p-coumglc trans, and Mv-3-p-coumglc trans. Mv-3-O-Glu (30.59–41.31 mg·L^-1^) was the most abundant species of the various monomeric anthocyanins and Dp-3-O-Glu (0.25–0.41 mg·L^-1^) was the least abundant, which was consistent with levels previously measured in Merlot wines. As shown in Fig. 3, all anthocyanin compounds were found at significantly higher levels in wine samples pretreated with 400 W ultrasound and low temperature. All anthocyanin compounds were found to be increased with an increase of ultrasound power with low temperature pretreatment. The results indicated that ultrasound combined with low temperature pretreatment effectively increased the content of each monomeric anthocyanin. Interestingly, we did not find that low temperature pretreatment improved the content of anthocyanins in wine as previously reported [41], as neither the content of total anthocyanins nor of monomeric anthocyanins increased by low temperature pretreatment alone.

### 3.4 Chromatic characteristics

As shown in Table 2, significant changes of a*, b*, c*, h*, and ΔE were observed in wine samples pretreated by ultrasound combined with low temperature. The a*, c*, and ΔE levels of the wine samples pretreated with low temperature alone were observed to be significantly higher than those of the other groups. The wine samples pretreated with 400 W ultrasound alone were found to have the significantly highest b*, and samples pretreated with 400 W and low temperature were found to have the significantly highest h*. A larger a* value indicates a redder color and a larger b* value indicates a yellower color. With ultrasound treatment, the higher the power, the redder the wine sample. Ultrasound treatment changed the color of the wine to yellow without low temperature pretreatment but changed the color of the wine sample to blue with low temperature pretreatment.

**Table 2:** Effects of ultrasound combined with low temperature pretreatment on the chromatic characteristics of Merlot wine.

	Ultrasonic power	Apparent appearance	a*	b*	c*	h*	**Δ**E
NLT	0 W		22.24± 0.02 g	7.23± 0.03e	23.38± 0.03 g	0.31± 0.01c	29.59± 0.04d
	120 W		24.45± 0.04d	7.50± 0.06d	25.61± 0.16d	0.30± 0.01c	31.72± 0.01c
	240 W		25.03± 0.04b	7.61± 0.06c	26.16± 0.06b	0.30± 0.01c	32.50± 0.01b
	400 W		24.73± 0.07c	8.16± 0.02a	25.97± 0.02c	0.32± 0.00b	32.73± 0.01ab
LT	0 W		25.23± 0.15a	7.71± 0.02b	26.51± 0.04a	0.30± 0.00c	33.27± 0.04a
	120 W		19.93± 0.06 h	6.97± 0.01 g	21.19± 0.06 h	0.34± 0.00a	26.37± 0.06e
	240 W		22.90± 0.04f	7.14± 0.02f	24.00± 0.03f	0.30± 0.00c	30.18± 0.30d
	400 W		23.82± 0.02e	5.53± 0.02 h	24.49± 0.04e	0.23± 0.00d	29.54± 0.80d

Note: Different letters in the row indicate significant differences (Duncan test, *P* ≤ 0.05) among treatments. NLT: No low temperature pretreatment: NT: Low temperature pretreatment.

### 3.5 Phenolic acid profile

Phenolic acids play an important role in controlling oxidation [42]. Phenolic compounds in wine samples pretreated by ultrasound combined with low temperature are listed in Table 3, expressed as the means (mg·L^-1^) of three analytical replicates. A total of 14 phenolic acid compounds were determined in the samples. The content of different phenolic acids also differed according to the pretreatment. The highest substance content was found in ultrasound combined with low temperature pretreated samples (except for protocatechuic acid, gentianic acid, quercetin, and kaempferol). Gallic acid, epicatechin, rutin, and resveratrol contents of wine samples pretreated by ultrasound combined with low temperature were positively correlated with the ultrasound power, while the content of caffeic acid was negatively correlated. The total amount of phenolic acid in the wine samples with ultrasound treatment was higher than that in the corresponding control group. In conclusion, ultrasound combined with low temperature pretreatment significantly increased the content of most phenolic acids and the total phenolic content.

**Table 3:** Effects of ultrasound combined with low temperature pretreatment on phenolic acid substances of Merlot wine (mg·L^-1^).

	Ultrasonic power								
	No-low temperature pretreatment					Low temperature pretreatment			
	0 W	120 W	240 W	400 W		0 W	120 W	240 W	400 W
Gallic acid	9.38± 3.41bc	8.78± 0.69bc	11.05± 1.56abc	10.78± 1.23abc		9.43± 4.15abc	7.30± 0.49c	12.94± 1.29ab	14.93± 0.34a
Protocatechuic acid	9.98± 1.45ab	14.90± 0.23a	6.39± 2.05b	8.56± 1.43ab		6.61± 3.16b	4.76± 0.08b	4.51± 0.03b	11.27± 7.40ab
Catechin	3.30± 0.02ab	2.84± 0.35ab	nd	1.85± 0.61b		4.22± 2.08a	4.50± 0.31a	3.93± 0.29a	2.54± 0.20ab
Gentianic acid	213.97± 16.63d	196.14± 4.02d	374.06± 39.63a	370.53± 6.42a		260.59± 8.29c	302.51± 3.51bc	271.45± 9.12bc	312.55± 22.98b
Caffeic acid	6.19± 3.20a	6.89± 1.88a	9.96± 0.70a	8.84± 0.25a		9.02± 1.01a	9.17± 1.44a	9.77± 0.66a	8.42± 0.20a
Epicatechin	11.22± 0.32d	12.31± 0.16 cd	11.97± 0.77d	11.10± 0.18d		11.28± 0.17d	13.74± 0.13b	13.70± 1.09bc	18.55± 0.81a
P-coumaric acid	9.44± 0.39ab	9.10± 0.01b	9.30± 0.04ab	9.32± 0.00ab		9.30± 0.10ab	9.33± 0.19ab	9.74± 0.17a	9.40± 0.31ab
Rutin	15.03± 2.71ab	12.30± 0.81b	12.77± 1.02b	12.03± 0.56b		14.19± 0.02ab	14.81± 1.44ab	16.20± 1.50a	17.43± 0.36a
Trans-ferulic acid	8.02± 1.85ab	5.86± 1.47b	7.48± 1.00ab	7.58± 0.00ab		8.52± 1.49ab	7.42± 1.12ab	9.50± 1.38a	6.84± 0.17ab
Rhizoside	3.38± 0.22ab	1.93± 0.76bcd	1.59± 1.00 cd	1.15± 0.62d		1.10± 1.00d	2.55± 0.55abcd	3.93± 0.48a	2.95± 0.34abc
Resveratrol	6.47± 0.01d	6.50± 0.02 cd	6.55± 0.02c	6.55± 0.00 cd		6.64± 0.02b	6.67± 0.03b	6.68± 0.03b	6.90± 0.08a
Quercetin	2.53± 0.05b	3.05± 0.12a	2.44± 0.08bc	2.39± 0.01bc		2.48± 0.07bc	2.46± 0.04bc	2.49± 0.02bc	2.37± 0.04c
Kaempferol	1.83± 0.02d	1.84± 0.01d	1.96± 0.00c	2.47± 0.00a		2.03± 0.06bc	2.01± 0.04bc	2.00± 0.02c	2.07± 0.03b
Gallic acid	9.38± 3.41bc	8.78± 0.69bc	11.05± 1.56abc	10.78± 1.23abc		9.43± 4.15abc	7.30± 0.49c	12.94± 1.29ab	14.93± 0.34a
Total	300.74	282.43	454.75	453.15		345.43	387.24	366.85	416.22

Note: Different letters in the row indicate significant differences (Duncan test, P ≤ 0.05) among treatments. “nd” indicates that the substance was not detected.

### 3.6 Antioxidant activities

Antioxidant activity and total phenolic content are highly correlated [43]. In this study, it was found that ultrasound combined with low temperature pretreatment affected the content of anthocyanins and phenolic acids in wine samples. Therefore, two methods (FRAP and DPPH) were further used to determine the antioxidant capacity of the wine samples, and the results are shown in Fig. 3. The highest values of FRAP appeared in the wine samples of the LT + U400 group (3.73 ± 0.05 U·mL^−1^), but the highest values of DPPH appeared in the LT + U240 group (50.23 ± 5.39 %), and both were also significantly higher than those of the CK group (3.17 ± 0.03 U·mL^−1^ and 22.23 ± 2.69 %, respectively). The values of FRAP showed that only ultrasound at the highest power (400 W) significantly increased the antioxidant capacity of the wine samples, but the values of DPPH showed that the antioxidant capacity of the wine samples was improved with any power of ultrasound treatment. The DPPH results showed that low temperature pretreatment improved the antioxidant capacity of the wine samples (except for 120 W ultrasound), and the effect was notable at medium and high ultrasound power, while FRAP results did not show this effect. The results of both methods showed that ultrasound combined with low temperature pretreatment contributed to improving the antioxidant activity of the wine samples, which was consistent with the effect on the content of anthocyanins and phenolic acids.

### 3.7 Volatile components

**Fig. 4:**
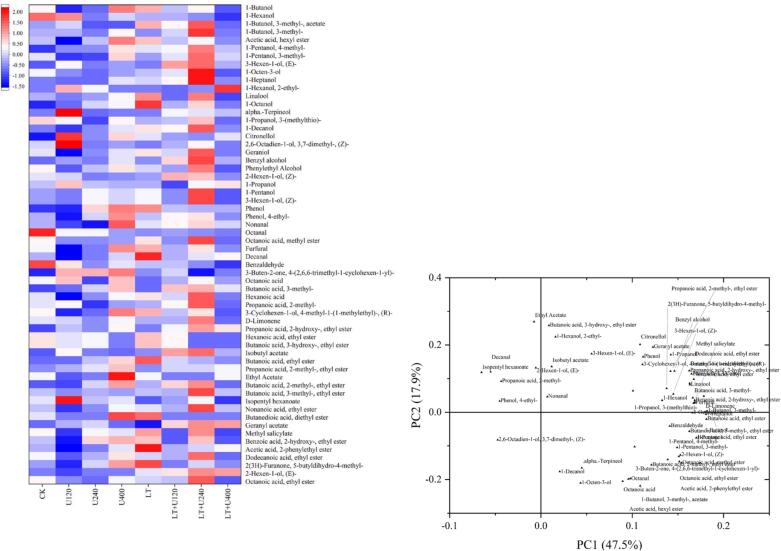
Effects of ultrasonic combined with low temperature pretreatment on volatile substances of Merlot wine. (A): Aroma substance heat map, (B): scores plot of PCA results for the reference samples of different treatment group. (C): loadings plot of PCA results for the reference samples of different treatment group. CK: No-low temperature pretreatment, U120: 120 W ultrasound treatment, U240: 240 W ultrasound treatment, U400: 400 W ultrasonic treatment, LT: low temperature pretreatment, LT + U120: low temperature pretreatment and 120 W ultrasound treatment, LT + U240: low temperature pretreatment and 240 W ultrasound treatment, LT + U400; low temperature pretreatment and 400 W ultrasound treatment.

**Fig. 5:**
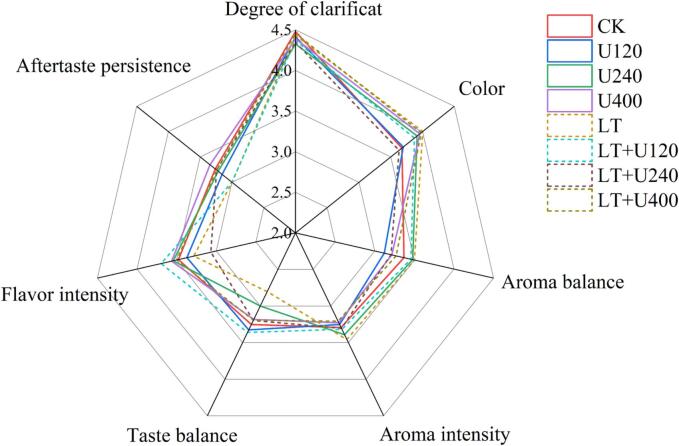
Effects of ultrasonic combined with low temperature pretreatment on sense of Merlot wine. CK: No-low temperature pretreatment, U120: 120 W ultrasound treatment, U240: 240 W ultrasound treatment, U400: 400 W ultrasonic treatment, LT: low temperature pretreatment, LT + U120: low temperature pretreatment and 120 W ultrasound treatment, LT + U240: low temperature pretreatment and 240 W ultrasound treatment, LT + U400; low temperature pretreatment and 400 W ultrasound treatment.

The volatile compounds were qualitatively and quantitatively analyzed by GC–MS. To more intuitively reflect the differences of volatile components in different wine samples, the content of the volatile components was normalized and then drawn into a heat map. The results are shown in Fig. 4A. Sixty volatile components were measured in the wine samples, including 37 alcohols, four fatty acids, 21 esters, two terpenes, and six carbonyl compounds. Higher alcohols have been related to pungent, caramel, and fruity odors [44], and esters affect the fruity aroma of wine [45]. In general, ultrasound combined with low temperature pretreatment increased the content of most alcohols and esters in the volatile components of the wine samples. Particularly when the ultrasound power was 240 W, the contents of most volatile components reached a maximum, indicating that the pretreatment improved the caramel and fruity odors of the wine. 1-Octanol and phenethyl acetate produce intense citrus and pleasant floral aromas, respectively [46], contributing positively to the wine aroma. Low temperature pretreatment increased the content of these two compounds and thus improved the aroma of the wine, but there were losses after combining with ultrasound, although the levels were still higher than those of the control group. Ultrasound treatment increased the content of caprylic acid, which imparts a rancid feeling to red wine [46], but low temperature pretreatment offset this effect. Combined treatment reduced benzaldehyde and 1-ethanol content to reduce the green and bitter almond taste [47]. Fig. 4B and 4C show the PCA data. Multivariate data analysis revealed the effects of combined treatment on the volatile components of the wine. The first two principal components accounted for 65.4 % (PC1 and PC2 were 47.5 % and 17.9 %, respectively). The distance between the LT and the CK groups on the load diagram was far, indicating that their volatile components were different, which was consistent with the results shown in the heat map (Fig. 5A). Combined with the score chart, it was found that the differences between the two groups were mainly in 1-octanol and octanoic acid. LT + 120U was also located far away from the other groups, which was primarily reflected by ethyl acetate levels. The difference between the experimental and CK groups in PC1 was mainly due to various esters. In conclusion, the combined treatment was beneficial to the formation of a pleasant aroma and greatly reduced undesirable aromas.

### 3.8 Sensory analysis

Fig. 5 shows the results of the sensory evaluation of 18 panelists on eight wine samples. There were no differences in the degree of clarification between the wines from different pretreatments, and all scored between 4.3 and 4.5 points. Low temperature pretreatment improved the wine aroma significantly, which increased the aroma intensity, and also increased the balance degree, but ultrasound treatment interfered with these improvements. In terms of taste, the wines with low temperature pretreatment did not perform well, except combined with 120 W ultrasound treatment. The wine samples treated with 400 W ultrasound had the highest aftertaste persistence scores, and the red wines that were not treated with low temperature had higher overall aftertaste scores. Ultrasound treatment of wine samples offset the negative effects of low temperature on the aftertaste. As such, ultrasound combined with low temperature treatment improved the aftertaste performance of red wine and offset part of the influence of low temperature on aroma and taste.

## 4 Conclusions

This study investigated the effects of combined ultrasound and low temperature pretreatment on the quality of dry red wine. The results showed that the effect of combined ultrasound and low temperature pretreatment on the fermentation process was mainly to increase the initial specific gravity and sugar concentration of the must. Combined pretreatment significantly increased the content of anthocyanins and phenolic acids while increasing the TPC, TAC, and TFC, which further affected the color of the wine and significantly improved the antioxidants. Furthermore, combined treatment affected the composition of volatile substances, thus affecting the aroma of the wine and reducing the generation of undesirable odors. Low temperature pretreatment improved the fruit and flower aroma of the wine, and combined ultrasound pretreatment offset the green taste induced by low temperature. In the sensory aspect, combined pretreatment contributed to improving the quality of the aftertaste. Pretreatment with 240 W ultrasound combined with low temperature increased the content of anthocyanins and phenols in wine and improved the aroma, thus demonstrating excellent application prospects.

## CRediT authorship contribution statement

**Qi Xie:** Writing – original draft, Visualization, Formal analysis. **Yurou Tang:** Investigation, Data curation. **Xueyan Wu:** Investigation, Data curation. **Qingyan Luo:** Investigation, Data curation. **Wentong Zhang:** Investigation, Data curation. **Hanyang Liu:** Investigation, Data curation. **Yulin Fang:** Writing – review & editing. **Xiaofeng Yue:** Conceptualization, Resources. **Yanlun Ju:** Supervision.

## Declaration of Competing Interest

The authors declare that they have no known competing financial interests or personal relationships that could have appeared to influence the work reported in this paper.
